# Decellularized Adipose Tissue Hydrogel Promotes Bone Regeneration in Critical-Sized Mouse Femoral Defect Model

**DOI:** 10.3389/fbioe.2019.00211

**Published:** 2019-09-06

**Authors:** Omair A. Mohiuddin, Brett Campbell, J. Nick Poche, Michelle Ma, Emma Rogers, Dina Gaupp, Mark A. A. Harrison, Bruce A. Bunnell, Daniel J. Hayes, Jeffrey M. Gimble

**Affiliations:** ^1^Center for Stem Cell Research and Regenerative Medicine, Tulane University School of Medicine, New Orleans, LA, United States; ^2^School of Medicine, Tulane University, New Orleans, LA, United States; ^3^School of Medicine, Louisiana State University, New Orleans, LA, United States; ^4^Obatala Sciences, New Orleans, LA, United States; ^5^Department of Biomedical Engineering, Tulane University, New Orleans, LA, United States; ^6^Department of Biomedical Engineering, Pennsylvania State University, State College, PA, United States; ^7^LaCell LLC, New Orleans, LA, United States

**Keywords:** critical-sized femoral defect, decellularized adipose tissue hydrogel, adipose derived stromal/stem cells (ASC), hydroxyapatite, histology, immunohistochemistry

## Abstract

Critical-sized bone defects fail to heal and often cause non-union. Standard treatments employ autologous bone grafting, which can cause donor tissue loss/pain. Although several scaffold types can enhance bone regeneration, multiple factors limit their level of success. To address this issue, this study evaluated a novel decellularized human adipose tissue (DAT) hydrogel as an alternative. In this study, DAT hydrogel alone, or in combination with adipose-derived stromal/stem cells (ASC), osteo-induced ASCs (OIASC), and hydroxyapatite were tested for their ability to mediate repair of a critical-sized (3 mm) femoral defect created in C57BL/6 mice. Micro-computed tomography results showed that all DAT hydrogel treated groups significantly enhanced bone regeneration, with OIASC + hydroxyapatite treated group displaying the most robust bone regeneration. Histological analyses revealed that all treatments resulted in significantly higher tissue areas with the relative mineralized tissue area significantly increased at 12 weeks; however, cartilaginous content was lowest among treatment groups with OIASC. Immunohistochemical analyses showed that DAT hydrogel enhanced collagen I and osteopontin expression, while the addition of OIASCs to the hydrogel reduced collagen II levels. Thus, DAT hydrogel promotes bone regeneration in a critical-sized femoral defect model that is further enhanced in the presence of OIASCs and hydroxyapatite.

## Introduction

Bones possess robust regenerative capacity allowing animals to repair fractures rapidly and effectively. However, “critical-sized defects” that exceed a threshold level can impair the response to growth factor-induced signal transduction mechanisms, leading to a failure in the natural healing process and subsequent non-unions (Clough et al., 2015a; Schemitsch, 2017). In humans, critical-sized defects can result from severe trauma, blast injuries, and large scale bone resection due to infection or tumors (Schemitsch, 2017). Some physiological factors that enhance the risk of non-unions include diabetes, obesity, inflammatory arthritis, and hypothyroidism (Nauth et al., 2018; Roddy et al., 2018). Orthopedic repair of critical-sized defects generally requires an invasive procedure that may include autologous or allogeneic bone grafting (Polo-Corrales et al., 2014). Autologous bone grafting is considered the gold-standard in critical-sized defect repair; however, the tissue source is limited, and the procedure often leads to prolonged donor site pain (Roddy et al., 2018; Huang et al., 2019). While allogeneic bone grafting does not face these same challenges, it carries the risk of eliciting a host immune response and infection transmission (Clough et al., 2015b; Ghassemi et al., 2018). Consequently, there remains an orthopedic demand for a biocompatible material that can replace or augment conventional autologous or allogeneic bone grafting.

An ideal bio-scaffold for bone regeneration would support osteogenesis, angiogenesis, and provide adequate space for infiltration of cells and nutrients (Roddy et al., 2018). Different types of scaffolds that have successfully promoted bone regeneration are ceramics, polymers (synthetic and natural) and decellularized/demineralized bone extracellular matrix (ECM) based scaffolds (Ghassemi et al., 2018; Roddy et al., 2018). Ceramic scaffolds including hydroxyapatite (HA) and tricalcium phosphate (TCP) are biocompatible and osteo-inductive, though they are brittle and poorly resorbed (Ghassemi et al., 2018; Roddy et al., 2018; Schmidt et al., 2019; Zhang et al., 2019). Synthetic polymers, such as polylactic acid (PLA) are bioresorbable with limited osteoinductive capability (Roddy et al., 2018). Ceramic/polymeric composite scaffolds have been found to possess enhanced strength and osteoinductive effect (Amini et al., 2012). Natural polymeric substances (e.g., collagen, alginate, etc.) display high biocompatibility and provide an excellent environment for cell attachment, but they lack adequate mechanical strength (Roddy et al., 2018). Decellularized and demineralized bone ECM provides a microenvironment which is biocompatible and osteoinductive; these products have also been shown to enhance regeneration in bone defect models *in vivo* (Shi et al., 2016; Yuan et al., 2017; Chen and Lv, 2018). Decellularized bone ECM presents a scaffold that is lower in immunogenicity and higher in mechanical strength than demineralized bone ECM (Liu and Lv, 2018). While demineralized bone ECM has proven to be a highly bioactive material since the decalcification process exposes osteogenic factors, its poor mechanical properties limit its use for the repair of load-bearing bones (Liu and Lv, 2018). Thus, while multiple scaffolds can improve bone regeneration in critical-sized defect models, there are factors limiting their level of success.

Adipose tissue extracted from humans undergoing lipectomy, abdominoplasty, or breast reduction is considered medical waste and is, therefore, an abundantly available tissue resource (Schneider et al., 2017). This makes adipose tissue an excellent candidate as a biomaterial for tissue engineering applications (Flynn, 2010; Song et al., 2018). Analysis of DAT has shown that it contains collagens (I, III, IV, VI, and VI), glycosaminoglycans (GAG), laminin, elastin, fibronectin, vascular endothelial growth factor (VEGF), and fibroblast growth factor (FGF) (Mohiuddin et al., 2019). In the past decade, decellularized adipose tissue (DAT) has been proven as a versatile scaffold, which is compatible with several different cell types and useful for a wide range of potential clinical applications (Mohiuddin et al., 2019). DAT promotes adipogenic differentiation of adipose stem cells (ASCs) *in vitro* (Turner et al., 2012; Yu et al., 2013; Brown et al., 2015) and is capable of recruiting and stimulating adipogenic differentiation of stem cells following subcutaneous implantation in rodents (Wang et al., 2013; Cheung et al., 2014; Han et al., 2015; Tan et al., 2017). ASCs seeded on DAT have also displayed osteogenic and chondrogenic differentiation when cultured in differentiation media (Choi et al., 2012; Guneta et al., 2017). Additionally, DAT has exhibited favorable clinical outcomes for wound healing (Woo et al., 2015), and nerve repair (Lin et al., 2011) in animal models.

Despite accumulating evidence supporting the potential of DAT scaffold for tissue regeneration, its use remains unexplored for the treatment of critical-sized bone defects. The present study evaluates a novel application of decellularized adipose tissue (DAT) as a bio-scaffold for the regeneration of critical-sized long bone defects. Based on prior studies, we postulated that DAT in combination with ASCs would support the regeneration of critical-sized femoral defects. Additionally, we postulated that the addition of either un-induced or osteoinduced ASCs with or without hydroxyapatite (HA) would augment the DAT hydrogel's regenerative potential. To test these hypotheses; a 3 mm femoral defect was created in immunocompetent mice and subsequently treated with different combinations of hydrogel with un-induced or osteoinduced ASCs (OIASCs) and HA.

## Materials and Methods

### Materials

All materials were purchased from Sigma-Aldrich (St. Louis, MO) or Fisher scientific (Waltham, MA) unless otherwise stated.

### Decellularized Adipose Tissue (DAT) Hydrogel Preparation

Adipose tissue was obtained with written informed consent from human donors (*n* = 3; females; BMI = 24.56 ± 2.52; Age = 50 ± 5.7, mean ± S.D.) undergoing elective lipectomy or abdominoplasty. The study protocol had been reviewed and approved by the Western IRB (Puyallup, WA) prior to implementation. Adipose tissue from the three donors was pooled together in equal ratio and decellularization was performed using a modification of the methods described by Flynn (2010), Zhang et al. (2016), and Thomas-Porch et al. (2018). The tissue was frozen/thawed three times for 1 h each, followed by washes in; deionized water for 48 h, 0.5 M and 1 M sodium chloride solution for 4 h each, deionized water overnight, 0.25% trypsin for 2 h, isopropanol for 48 h, deionized water overnight, 1% triton X-100 for 48 h and deionized water for 48 h. DAT was lyophilized for 48 h and cryo-milled into a powder.

DAT hydrogel was prepared using the method previously described by Young et al. (2011), and Tan et al. (2017). First, pre-hydrogel was prepared by digesting 50 mg of DAT powder in 1 mL of pepsin solution (10 mg pepsin dissolved in 1 mL 0.05 N HCl) for 48 h. Immediately before use the pre-hydrogel was neutralized with 1 M NaOH and incubated at 37°C for 30 min to form a stable hydrogel.

### ASC Seeding, Culture, and Osteo-Induction in DAT Hydrogel

ASCs and media formulations were obtained from LaCell LLC (New Orleans, LA) and equal number of ASCs from all donors (*n* = 3; different from the adipose tissue donors) were pooled together, Passage 2-3 ASCs were used for all experiments. ASCs were suspended in pre-hydrogel at a concentration of 0.5 million cells per 100 μL and incubated at 37°C for 30 min. The hydrogel was subsequently submerged in stromal media for 2 days or osteogenic media (10 nM dexamethasone, 20 mM β-glycerophosphate, and 50 μM L-ascorbic acid) for 8 days for osteo-induction prior to implantation in mice.

### Experimental Animals/IACUC

All animal experiments were performed in accordance with a protocol reviewed and approved by Tulane University Institutional Animal Care and Use Committee (IACUC). 8-12-week-old C57BL/6 mice (male and female) were used for all femoral defect experiments.

### Femoral Defect Model

**Figure 1:**
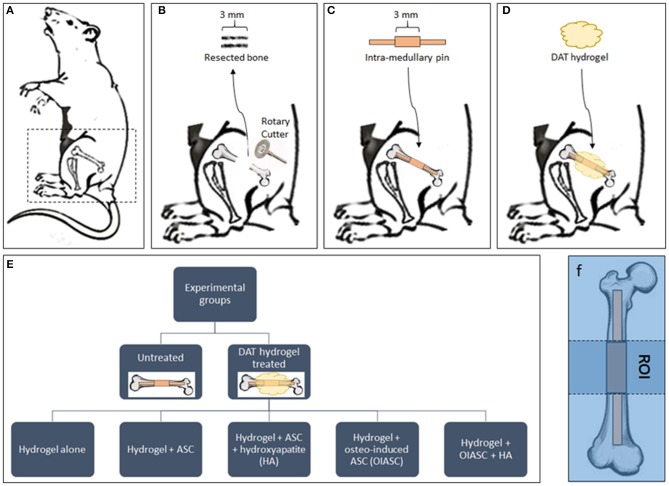
Diagrammatic representation of the experimental design. **(A)** Mice were placed in the right lateral position for surgery and all surgeries were performed on the left femur. **(B)** A 3 mm bone section was resected from the femoral diaphysis using a diamond-coated rotary cutter tool to create a critical-sized defect. **(C)** A pre-fabricated stainless-steel pin was inserted in the distal and proximal medullary cavity to establish a stable 3 mm gap between the two ends of the bone. The mice were left untreated as controls **(C)** or treated by the implantation of DAT hydrogel on the site of defect **(D)**. **(E)** The study included six experimental groups (one untreated and five hydrogel treated). **(F)** The region of interest (ROI) for all subsequent analyses was defined as the area between the proximal and distal ends of the collar (central 3 mm of the medullary pin).

The surgical procedure was based on the method described by Clough et al. (2015a). Mice were anesthetized with 3% isoflurane (VET one; Boise, ID) and were pre-operatively injected with 0.1% buprenorphine (Reckitt Benckiser; Slough, UK) for analgesia. The surgical site was first shaved and cleaned with povidone/iodine solution. The skin on the left thigh was incised longitudinally along the femur from the knee to the hip joint to expose the underlying muscle. An incision was made along the white line where the septa of both the muscles meet to visualize the bone shaft (Figure 1A). A periosteal elevator was used to expose the central portion of the bone. The central 3 mm section was marked on the bone diaphysis and resected using a diamond-coated rotary cutter tool (Strauss diamond 361.514.080HP; Palm coast, FL) with saline irrigation (Figure 1B). A pre-fabricated stainless-steel medullary pin was inserted in the distal and proximal medullary cavity to establish a stable 3 mm gap between two ends of the bone (Figure 1C).

Mice were divided in to six experimental groups (*n* = 8 per group; *n* = 4 males, and *n* = 4 females): Untreated; hydrogel treated; hydrogel + ASC treated; hydrogel + ASC + hydroxyapatite (HA) treated; hydrogel + osteo-induced ASC (OIASC) treated; hydrogel + OIASC + HA treated (Figure 1E). To make HA-hydrogel composite 50 mg of HA was added to the pre-hydrogel and the mixture was vortexed to ensure uniform distribution. Following hydrogel implantation (Figure 1D), the skin was sutured and recovery of the mice from anesthesia was monitored. Mice were injected with 0.1% buprenorphine every 12 h for up to 3 days post-operatively. Half of each experimental group was harvested at two time points, either 6 or 12 weeks. Animals were euthanized by carbon dioxide asphyxiation followed by cervical dislocation. Femurs were removed and fixed in 10% buffered formalin until further analysis.

### Micro-Computed Tomography (μCT)

All μCT scans were performed using Skyscan 1172 unit (Bruker; Billerica, MA). After data acquisition, axial images were reconstructed using NRecon software (Skyscan; Billerica, MA) and subsequently analyzed with CTAn software (Skyscan; Billerica, MA).

The femurs were positioned in vertical orientation and scanned at; 100 KV, 100 μA, 8 μm resolution, Cu/Al filter, 360° scan with 0.2° rotation step, 1,553 ms exposure time, frame averaging (10), random movement (10), and flat field and geometrical correction “ON.” The images were thresholded to subtract the pin from the bone and the region of interest (ROI) was defined as the area between the proximal and distal ends of the collar (central 3 mm of the medullary pin) (Clough et al., 2015b; Figure 1F). Regenerated bone volume, bone area, trabecular thickness, and polar moment of inertia (PMI) were determined within the ROI using three-dimensional analysis in CTAn software as described by Clough et al. (2015b).

### Histology and Immunohistochemistry (IHC)

Formalin fixed femurs were decalcified using Immunocal® for 24 h, then paraffin embedded with the intramedullary pin in place. Axial sections (5 μm) of the bone were cut above the intra-medullary pin. Tissue sections were stained with hematoxylin and eosin (Richard Allan Scientific; San Diego, CA), Masson's trichrome, and safranin O and fast green (Scy Tek Lab; Logan, UT), followed by dehydration in graded ethanol solutions and sealing with Permount mounting medium. Two non-consecutive sections for each sample were used for analysis and quantification.

For IHC staining tissue sections were de-paraffinized in xylene, hydrated in graded ethanol solutions, endogenous peroxidases were blocked by 3% hydrogen peroxide treatment for 15 min and antigen retrieval was performed in citrate buffer at 95°C for 20 min. Samples were incubated overnight with primary antibodies against collagen I (1:10 dilution, mouse monoclonal, 8-3A5, DSHB, Iowa city, IA), osteopontin (1:50 dilution, mouse monoclonal, MPIIIB10(1), DSHB, Iowa city, IA), collagen II (1:400, rabbit polyclonal, ab34712, Abcam, Cambridge, MA) and KI67 (1:400, rabbit polyclonal, ab15580, Abcam, Cambridge, MA). Samples were then incubated with HRP tagged secondary antibody (1:500, goat anti-mouse, 31430, ThermoFisher, Waltham, MA; 1:500, goat anti-rabbit, ab6721, Abcam, Cambridge, MA), followed by ImmPACT® DAB Peroxidase (HRP) Substrate (VectorLabs; Burlingame, CA). Finally, the samples were counter stained with hematoxylin, dehydrated, and sealed.

### Image Processing

Histological and IHC Images were acquired with Axio Scan.Z1 slide scanner (Zeiss; Oberkochen, Germany) and analyzed with ImageJ software. ROIs were selected manually, and color deconvolution tool was used to quantify the stains. Hematoxylin and eosin (H&E) stained sections were used to determine the total area of regenerated tissue (pink + purple color), Masson's trichrome (MT) for mineralized tissue area (% blue color; blue = mineralized tissue, red = osteoid), and safranin O (SO) for cartilage area (% red color; red = cartilage, green = bone) (**Figures 4**, **5**). For IHC analysis protein antigen signal was quantified by DAB stain (% brown color; brown = protein of interest, blue = background) (**Figures 6**, **7**). All quantifications are presented as relative values normalized to the untreated group as a control.

### Statistics

Statistical analysis and plotting of data were performed using Prism 5 (GraphPad, San Diego, CA). All the results are expressed as mean ± standard deviation (SD). Normality of the data were determined by Shapiro-Wilk test. Statistical analysis of μCT data was performed using *t*-test and Mann-Whitney test, while histochemical and IHC data were analyzed using paired *t*-test and Wilcoxin matched pair test. The *p*-values <0.05 were designated as statistically significant for all results.

## Results

### Gross Anatomy of Regenerated Femurs

**Figure 2:**
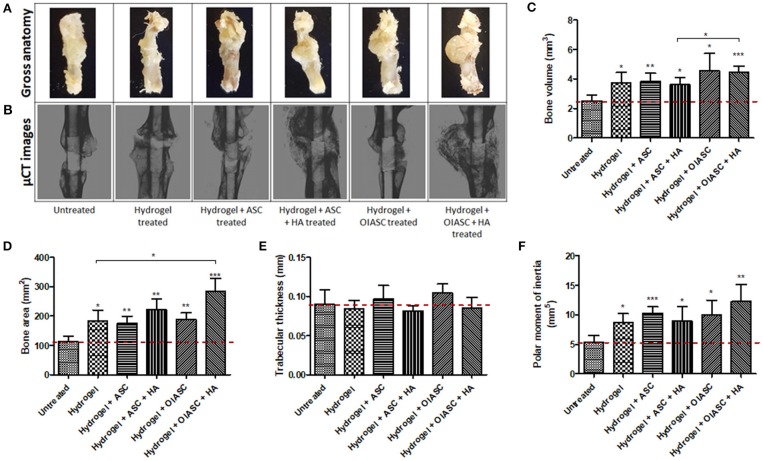
Six weeks post-surgery comparison of bone regeneration between the untreated and DAT hydrogel treated groups using μCT. Representative gross anatomy **(A)** and μCT scan **(B)** images of untreated and treated femurs are displayed. Measurements were made of regenerated bone volume **(C)**, bone area **(D)**, trabecular thickness **(E)**, and polar moment of inertia **(F)** using reconstructed axial μCT images. Data are expressed as mean (*n* = 4) ± SD; level of significance: (*) *p* < 0.05; (**) *p* < 0.01; (***) *p* < 0.001.

**Figure 3:**
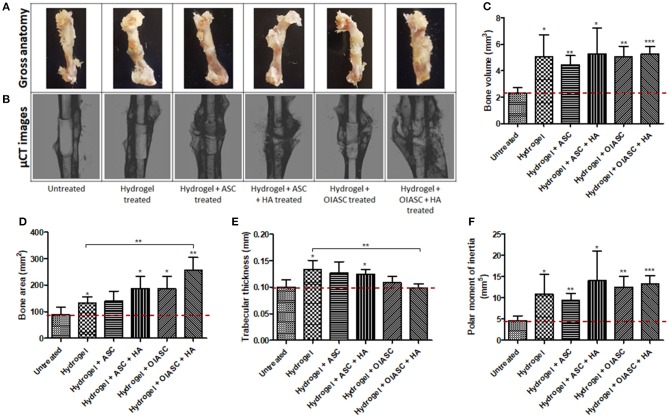
Twelve weeks post-surgery comparison of bone regeneration between the untreated and DAT hydrogel treated groups using μCT. Representative gross anatomy **(A)** and μCT scan **(B)** images of untreated and treated femurs are displayed. Measurements were made of regenerated bone volume **(C)**, bone area **(D)**, trabecular thickness **(E)**, and polar moment of inertia **(F)** using reconstructed axial μCT images. Data are expressed as mean (*n* = 4) ± SD; level of significance: (*) *p* < 0.05; (**) *p* < 0.01; (***) *p* < 0.001.

Gross analysis of the regenerated bones revealed that by week 6 after surgery, the intra-medullary pin was concealed by newly formed tissue and was no longer visible. In all samples, a distinct callus was visible around the region of the defect, while the callus was more prominent in the *hydrogel* + *ASC* + *HA, hydrogel* + *OIASC* and *hydrogel* + *OIASC* + *HA* treated groups (Figure 2A). At 12 weeks post-surgery, the callus was found to be significantly reduced in all *hydrogel treated* groups, and the physical appearance approached the dimensions of the original bone structure (Figure 3A).

### Analysis of Regenerated Bone by μCT

#### 6 Weeks

Six weeks after surgery, the femurs were collected and analyzed by μCT (Figure 2B). Quantification of bone volume, bone area, trabecular thickness, and PMI was performed around the 3 mm central region of the intramedullary pin (Figure 1F). All *hydrogel treated* groups were found to have attained significantly higher bone volume than the *untreated* group, with *hydrogel* + *OIASC* and *hydrogel* + *OIASC* + *HA treated* groups displaying the highest bone volume at 6 weeks after surgery (Figure 2C). Additionally, *hydrogel* + *OIASC* + *HA treated* group had significantly higher bone volume than the *hydrogel* + *ASC* + *HA treated* group (Figure 2C). Bone area (Figure 2D) and PMI (Figure 2F) were significantly higher for all *hydrogel treated* groups relative to the *untreated* group, while the *hydrogel* + *OIASC* + *HA treated* group had significantly higher bone area than the *hydrogel treated* group (Figure 2D). Among all *hydrogel treated* groups, *hydrogel* + *OIASC* + *HA treated* had the highest bone area and PMI, which displayed a more than 2-fold increase relative to the *untreated* group (Figures 2D,F). No significant variation was observed in trabecular thickness of the regenerated bone at 6 weeks between the *untreated* and *hydrogel treated* groups (Figure 2E).

#### 12 Weeks

Similar to the findings at 6 weeks, bone volume was significantly higher for all *hydrogel treated* groups compared to the *untreated* group at 12 weeks after surgery (Figure 3C); however, there were no significant differences in bone volume between the *treated* groups themselves (Figure 3C). All *hydrogel treated* groups displayed significantly higher bone area relative to the *untreated* group (Figure 3D), with *hydrogel* + *OIASC* + *HA treated* group presenting significantly higher bone area than the *hydrogel treated* group (Figure 3D). *Hydrogel, hydrogel* + *ASC*, and *hydrogel* + *ASC* + *HA treated* groups displayed higher trabecular thickness relative to the *untreated* group (Figure 3E). PMI was found to be significantly higher than the *untreated* group for all *hydrogel treated* groups, while there were no statistical differences between the *treated* groups (Figure 3F).

Comparison of bone regeneration between the 6- and 12-week time points revealed that; the *untreated* group showed no significant increment in bone volume at 12 weeks as compared to 6 weeks, while all *hydrogel treated* groups continued to enhance bone volume (though not significant) during the 6 and 12-week periods (Figure S1a). The bone area was insignificantly lower at 12 weeks than 6 weeks for all the experimental groups except *hydrogel* + *OIASC treated* group, yet the overall trend did not change between the two time-points (Figure S1b). The *hydrogel* and *hydrogel* + *ASC* + *HA treated* groups displayed a significant increase in trabecular thickness from 6 to 12 weeks (Figure S1c). In the HA containing *treated* groups most of the HA appeared to have incorporated in the developing bone callus at 6- and 12-weeks post-surgery (Figures 2B, 3B), however some particles were still visible around the callus which were excluded from the measurement.

### Histological Assessment of the Regenerated Tissue Area and the Mineralized/Cartilaginous Content

#### 6 Weeks

**Figure 4:**
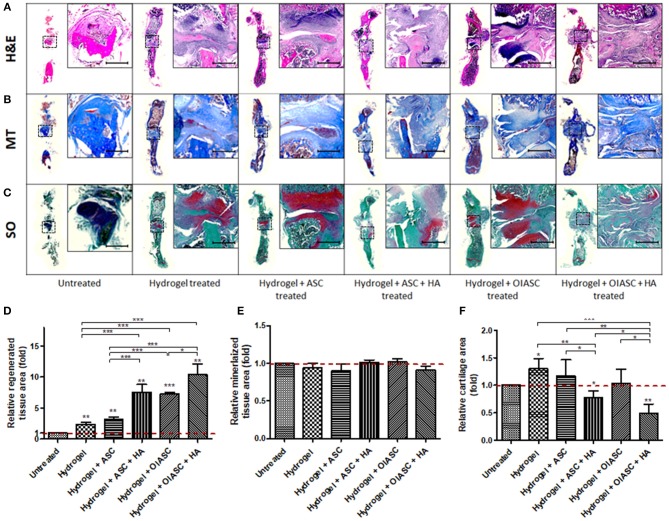
Histological analysis of demineralized bone at 6 weeks after surgery. Paraffin embedded sections were stained with H&E **(A)**, Masson's Trichrome (MT) **(B)**, and Safranin O (SO) **(C)** to determine tissue area **(D)**, mineralized tissue area **(E)**, and cartilage area **(F)**, respectively. **(A–C)** Representative images of stained axial sections (left in each panel) and magnified view of the ROI (right in each panel; scale bar 500 μm). **(D)** Regenerated tissue area was calculated as the sum of pink and purple color in H&E stained images **(A)**. **(E)** Mineralized tissue area was determined by the quantification of blue color in MT stained images **(B)**. **(F)** Cartilage area was determined by the quantification of red color in SO stained images **(C)**. All quantifications are presented as relative values normalized to the untreated group as a control. Data are expressed as mean (*n* = 4) ± SD; level of significance: (*) *p* < 0.05; (**) *p* < 0.01; (***) *p* < 0.001.

Histological analysis of demineralized bone was performed by the quantification of H&E, MT, and SO staining to determine tissue area, mineralized tissue area, and cartilage area respectively, using ImageJ software. Regenerated tissue area was calculated as the sum of pink and purple color in H&E stained images (Figure 4A), mineralized tissue area was determined by the quantification of blue color in MT stained images (Figure 4B), and cartilage area was determined by the quantification of red color in SO stained images (Figure 4C). Significantly higher tissue area was observed in all *hydrogel treated* groups relative to the *untreated* group (Figure 4D). Among *treated* groups *hydrogel* + *OIASC* + *HA* produced the highest amount of tissue, followed by *hydrogel* + *ASC* + *HA* and *hydrogel* + *OIASC treated* groups, whereas *hydrogel* and *hydrogel* + *ASC treated* groups exhibited the lowest tissue area (Figure 4D). At 6 weeks, no significant difference was observed in the relative mineralized tissue area between the groups (Figure 4E). Relative cartilage area within the regenerated tissue was found to be significantly higher in *hydrogel* and *hydrogel* + *ASC treated* groups as compared to the *untreated* group (Figure 4F). The *hydrogel treated* bones presented significantly higher cartilage area than all other experimental groups, whereas both *HA treated* groups displayed significantly lower relative cartilage area than other treated groups (Figure 4F).

#### 12 Weeks

**Figure 5:**
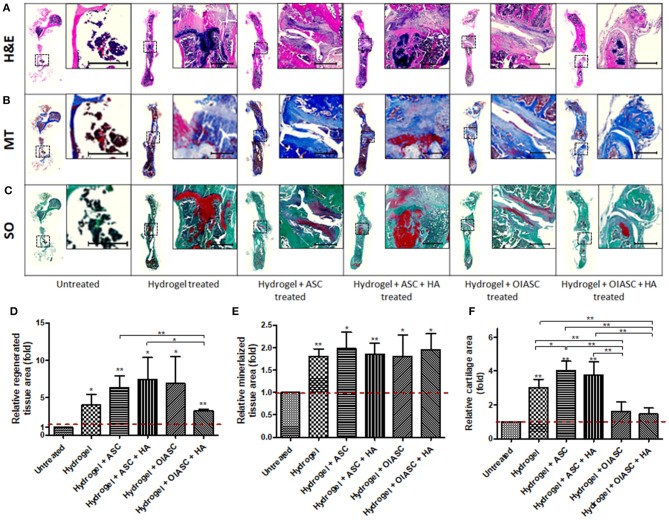
Histological analysis of demineralized bone at 12 weeks after surgery. Paraffin embedded sections were stained with H&E **(A)**, MT **(B)**, and SO **(C)** to determine tissue area **(D)**, mineralized tissue area **(E)**, and cartilage area **(F)**, respectively. **(A–C)** Representative images of stained axial sections (left in each panel) and magnified view of the ROI (right in each panel; scale bar 500 μm). ImageJ software was used for quantification of the stain area. **(D)** Regenerated tissue area was calculated as the sum of pink and purple color in H&E stained images **(A)**. **(E)** Mineralized tissue area was determined by the quantification of blue color in MT stained images **(B)**. **(F)** Cartilage area was determined by the quantification of red color in SO stained images **(C)**. All quantifications are presented as relative values normalized to the untreated group as a control. Data are expressed as mean (*n* = 4) ± SD; level of significance: (*) *p* < 0.05; (**) *p* < 0.01; (***) *p* < 0.001.

At 12 weeks all the *hydrogel treated* groups were found to have significantly higher tissue area relative to the *untreated* groups (Figure 5D). Likewise, relative mineralized tissue area was significantly higher for all *treated* groups relative to the *untreated* group (Figure 5E). *The hydrogel, hydrogel* + *ASC* and *hydrogel* + *ASC* + *HA treated* groups displayed significantly higher relative cartilage area as compared to the *untreated, hydrogel* + *OIASC treated*, and *hydrogel* + *OIASC* + *HA treated* groups (Figure 5F).

Comparative analysis of histological features within groups showed that the *hydrogel* and *hydrogel* + *ASC treated* groups continued to increase tissue area from 6 to 12 weeks, while there was a significant reduction in tissue area in both *OIASC treated* groups (Figure S2a). Mineralized tissue area decreased significantly in *untreated* and *hydrogel* + *OIASC treated* group from 6 to 12 weeks, although there was no significant variation within the other groups (Figure S2b). Cartilage area reduced in all experimental groups (though not significant), except in the samples treated with *hydrogel* + *ASC* + *HA*, which displayed an increase in cartilage area (Figure S2c).

### Immunohistochemical (IHC) Analysis of Regenerated Tissue ECM and Cell Proliferation

#### 6 Weeks

**Figure 6:**
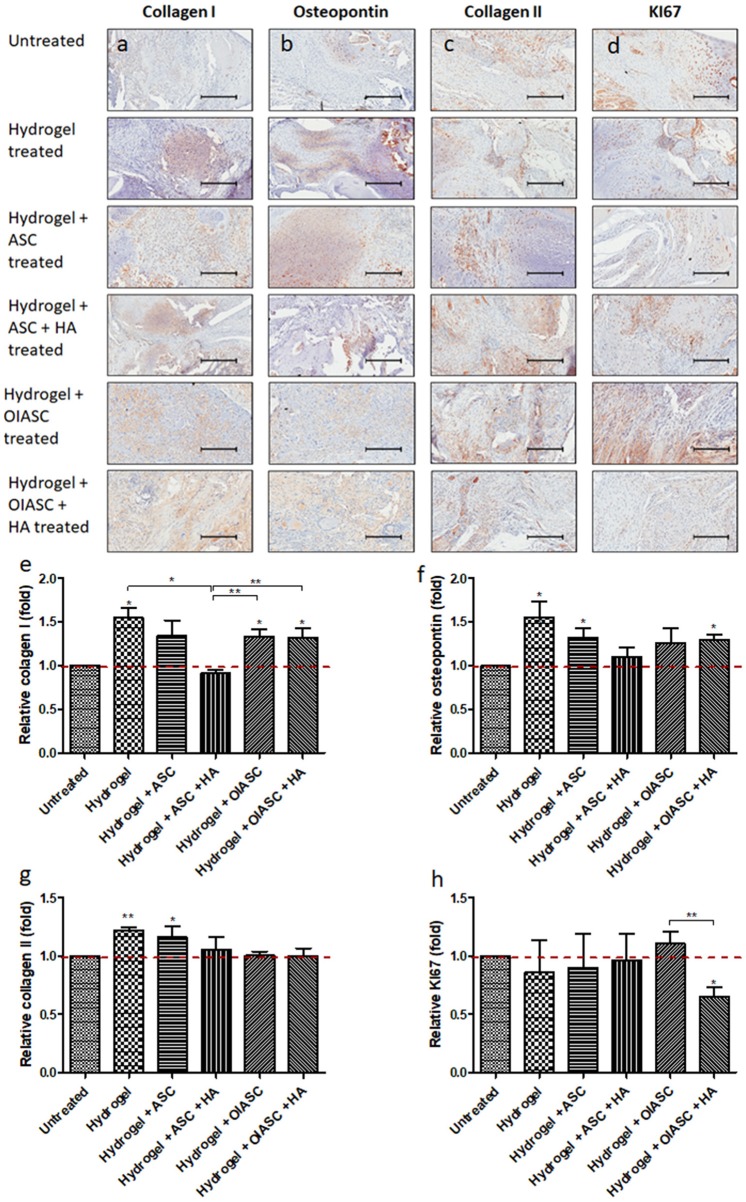
Analysis of the composition of regenerated bone ECM and cell proliferation at 6 weeks after surgery using immunohistochemistry. Paraffin embedded sections were stained with primary antibodies against collagen I **(a)**, osteopontin **(b)**, collagen II **(c)**, and KI67 **(d)**; and HRP tagged secondary antibody. The signal was visualized by HRP/DAB reaction and the sections were counterstained with hematoxylin. Quantification of the stained area (brown color) was performed to determine level of collagen I **(e)**, osteopontin **(f)**, collagen II **(g)**, and KI67 **(h)**. All quantifications are presented as relative values normalized to the untreated group as a control. Data are expressed as mean (*n* = 4) ± SD; level of significance: (*) *p* < 0.05; (**) *p* < 0.01; scale bar 200 μm.

The composition of regenerated ECM and cell proliferation was analyzed using IHC. Collagen I and osteopontin were used as markers of osteoblast activity, collagen II indicated the amount of cartilaginous tissue in regenerated bone, while KI67 reflected cell proliferation. All *hydrogel treated* groups resulted in the significantly higher deposition of collagen I in the tissue relative to the *untreated* group, except for the *hydrogel* + *ASC* + *HA treated* group which displayed significantly lower collagen I level than the *hydrogel, hydrogel* + *OIASC*, and *hydrogel* + *OIASC* + *HA treated* groups (Figure 6e). Osteopontin deposition was found to be significantly higher than the *untreated* group in *hydrogel, hydrogel* + *ASC*, and *hydrogel* + *OIASC* + *HA treated* group (Figure 6f). Collagen II levels were significantly higher in *hydrogel* and *hydrogel* + *ASC treated* groups relative to the *untreated group* (Figure 6g). Quantification of KI67 staining revealed that *hydrogel* + *OIASC* + *HA treated* samples had significantly fewer proliferating cells relative to the *untreated* and *hydrogel* + *OIASC treated* samples (Figure 6h). Apart from that, no significant variations were observed in cell proliferation among other groups (Figure 6h).

#### 12 Weeks

**Figure 7:**
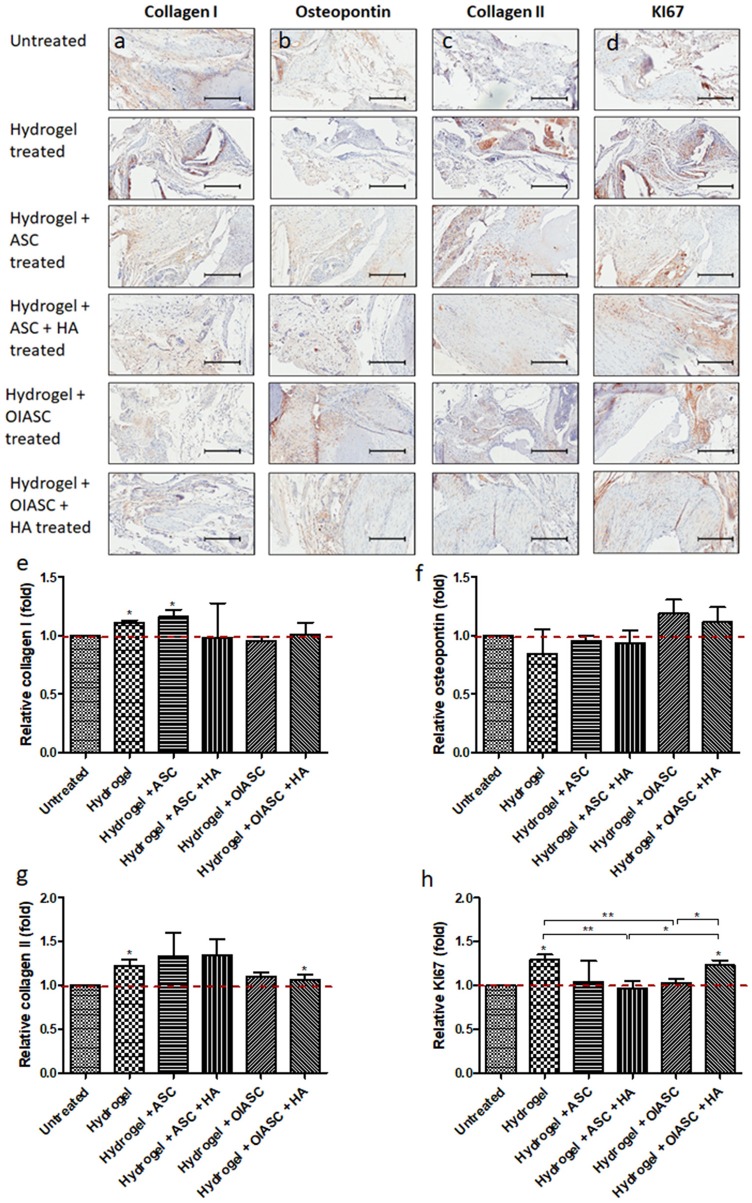
Analysis of the composition of regenerated bone ECM and cell proliferation at 12 weeks after surgery using immunohistochemistry. Paraffin embedded sections were stained with primary antibodies against collagen I **(a)**, osteopontin **(b)**, collagen II **(c)**, and KI67 **(d)** and HRP tagged secondary antibody. The signal was visualized by HRP/DAB reaction and the sections were counterstained with hematoxylin. Quantification of the stain area (brown color) was performed to determine level of collagen I **(e)**, osteopontin **(f)**, collagen II **(g)**, and KI67 **(h)**. All quantifications are presented as relative values normalized to the untreated group as a control. Data are expressed as mean (*n* = 4) ± SD; level of significance: (*) *p* < 0.05; (**) *p* < 0.01; scale bar 200 μm.

Significantly higher levels of collagen I were observed in *hydrogel* and *hydrogel* + *ASC treated* groups relative to the *untreated* group (Figures 7a,e). No significant differences were observed in relative osteopontin levels among all experimental groups, although both *OIASC* treated groups displayed higher relative osteopontin deposition than the *untreated* group (Figures 7b,f). Relative collagen II levels were found to be higher in *hydrogel treated* group than the *untreated* group (Figures 7c,g). Additionally, cell proliferation was significantly higher in *hydrogel* and *hydrogel* + *OIASC* + *HA treated* group relative to the *untreated* group (Figures 7d,h).

Comparative immunohistochemical analysis from 6 to 12 weeks, indicated no significant changes in collagen I and osteopontin levels within the *hydrogel treated* groups, however, a significant increase was observed in the *untreated* group (Figures S3a,b). A significant increase in collagen II level was observed in both the *ASC* treated groups (Figure S3c). Cell proliferation did not change significantly for any of the experimental groups, except *hydrogel* + *OIASC* + *HA* treated group, which displayed a significant increase in KI67 signal (Figure S3d).

## Discussion

The current study employed an immunocompetent mouse critical-sized femoral defect model to test the hypotheses that DAT hydrogel will promote regeneration of critical-sized femoral defect and that the addition of ASC, OIASC, and HA will enhance the regenerative capacity of DAT hydrogel. Bone regeneration was analyzed quantitatively by μCT, histology, and IHC.

The physical appearance of regenerated bones indicated higher tissue regeneration in the *hydrogel treated* groups as compared to the *untreated* group at 6 weeks after surgery, which suggested a higher regenerative activity. The μCT data validated our hypotheses that DAT hydrogel promoted bone regeneration, and the addition of OIASC and HA to the hydrogel resulted in higher bone volume and area than hydrogel alone; however, the addition of un-induced ASCs to the hydrogel did not enhance bone regeneration. At 12 weeks post-surgery, the bones appeared to have undergone significant anatomical remodeling and were returning to an intact physiological bone-like morphology. Additionally, the critical-sized defects bridged in all the *hydrogel treated* groups, while the *untreated* group failed to bridge the defect from all sides, thereby confirming the creation of a critical-sized defect. Among *hydrogel treated* groups, the *hydrogel* and *hydrogel* + *ASC treated* groups appeared the closest to being completely restructured after 12 weeks of treatment, while in the groups containing OIASC and/or HA a reduced callus was still visible. This finding was also confirmed by the μCT data, which showed that all *hydrogel treated* groups had acquired similar bone volumes by week 12 post-surgery, yet the bone surface area was still higher for *OIASC* and/or *HA* treated groups within the ROI. Additionally, *OIASC* and/or *HA* treated groups were found to possess a higher resistance to torsional force, based on the higher PMI values determined by μCT. PMI provides a rough estimate of torsional rigidity by determining the distribution of bone around the center of the specimen, it is inversely related to shear stress created in the bone (Schoenau et al., 2001; Bagi et al., 2006; Mejia et al., 2019).

Following μCT analysis, the bones were decalcified, paraffin embedded and sectioned for histological and IHC examination. Analysis of regenerated tissue area after 6 weeks of surgery was performed using H&E staining, which provided an estimate of the callus size in a 2-dimensional plane; however, it does not approximate the total regenerated tissue area. The histological results obtained for regenerated tissue area at 6 weeks post-surgery corresponded to the μCT results, where all *hydrogel treated* groups displayed significantly higher tissue area relative to the *untreated* group. Although μCT data had indicated differences in bone volume between experimental groups, no significant variation in percent mineralized area was observed, which indicated that the ratio of mineralized to non-mineralized tissue area was unaffected by the treatment. Combined analysis of tissue area at 6- and 12-week time points revealed that in the presence of OIASC and/or HA, the hydrogel rapidly promotes tissue regeneration and then enters the remodeling phase, whereas the hydrogel and ASC treated bones continue ECM deposition beyond the 6-week time point. Cartilage area was determined by the quantification of glycosaminoglycan content using SO staining. The presence of OIASC and/or HA in the hydrogel was found to diminish tissue cartilage content as compared to other *hydrogel treated* groups at both 6- and 12-weeks post-surgery.

At 6 weeks after surgery, it was found that except the *hydrogel* + *ASC* + *HA treated* group, all *hydrogel treated* groups displayed significant higher osteoblast activity, based on the higher collagen I and osteopontin expression determined by IHC. There was no significant variation in osteoblast activity within the *hydrogel treated* groups from 6 to 12 weeks after surgery; however, a significant increase was observed in the *untreated* group. This indicated that all the *hydrogel treated* groups resulted in earlier activation of osteoblasts, which consequently led to the higher bone volume as compared to the *untreated* group as observed by μCT analysis. Quantification of collagen II deposition was used to determine the cartilaginous content of regenerated tissue, which was found to be lower in the *OIASC treated* groups as compared to the other *hydrogel treated* groups at both time points. The low GAG and collagen II content in *OIASC treated* groups indicates a lower tendency to form cartilage than hydrogel alone or in combination with ASCs.

Collective examination of the μCT, histological and IHC data reveals that by week-12 after surgery, all *hydrogel treated* groups had attained similar bone volume (μCT analysis) and percent mineralized tissue area (histological analysis), which suggests that they had mineralized to a comparable extent. Among the *hydrogel treated* groups, the *OIASC treated* samples displayed the highest bone volume at 6 weeks after surgery, demonstrating an accelerated regenerative process as compared to other groups. Moreover, the *OIASC treated* samples were found to have considerably lower levels of cartilaginous content in the regenerated tissue relative to the other *hydrogel treated* groups, as evidenced by reduced deposition of both GAG and collagen II (Table 1). The lower cartilage content of *OIASC treated* samples indicates higher bone quality in terms of structure and mechanical strength, which was also confirmed by the PMI readings obtained from μCT. Based on these findings it can be inferred that in the absence of *OIASCs* endochondral ossification dominated the regenerative process, however, the presence of *OIASCs* minimized the intermediary chondrogenic phase of regeneration, thus expediting the healing process. In future, analysis of collagen X in regenerating tissue would provide greater insight on the effect of different treatments on the transition from cartilage to bone in the endochondral ossification process.

**Table 1:** Summary of the contribution of each treatment group to the two main components of healing process (bone and cartilage).

**Treatment group**	**Regenerative response**			
	**Bone**		**Cartilage**	
	**6 weeks**	**12 weeks**	**6 weeks**	**12 weeks**
Hydrogel alone	↑	↑	↑*↑↑*	↑↑
Hydrogel + ASC	↑↑	↑↑	↑*↑↑*	↑↑
Hydrogel + ASC + HA	↑	↑	↑↑	↑↑
Hydrogel + OIASC	↑	↑↑	↑↑	↑
Hydrogel + OIASC + HA	↑*↑↑*	↑*↑↑*	↑	↑

*Arrows indicate qualitative extent of regeneration relative to the untreated control. Qualitative assessment of bone regeneration is based on cumulative μCT data (bone volume and area), while cartilage regeneration is based on histochemical (Safranin O stain), and IHC (Collagen II) analysis.

Several recent studies have focused on the development of hydrogel-based scaffolds to replace bone grafting for the treatment of critical-sized femoral defect. Some of the scaffolds tested for this purpose thus far include alginate (Smith et al., 2014b), hyaluronan (Hulsart-Billstrom et al., 2015; Barbeck et al., 2016), calcium phosphate cement (Ahlfeld et al., 2017), and polyethylene glycol (PEG) (Sonnet et al., 2013) based hydrogels. Additionally, the hydrogels were supplemented with growth factors like VEGF, TGF-β, and BMP2 to enhance their osteoconductive capacity (Sonnet et al., 2013; Smith et al., 2014a,b). Our data shows that DAT hydrogel alone is capable of promoting bone regeneration, potentially due to the presence of growth factors that are inherently present in decellularized adipose ECM as shown before (Brown et al., 2011; Wang et al., 2013; Shi et al., 2016). Bone marrow-derived mesenchymal stem cells (BMSCs) have been the cells of choice for most bone regeneration studies published in the recent years (Clough et al., 2015b; Strong et al., 2016; Robey, 2017; Nau et al., 2018). We elected to use ASCs for our experiments due to their proven capability to undergo osteogenic differentiation, easier availability, and consequent translatability to human subjects (Levi and Longaker, 2011). Our findings show that the addition of OIASCs was able to enhance the regenerative potential of DAT hydrogel. This outcome is consistent with the results published by Clough et al. (2015b) who used osteoinduced BMSCs for their experiments. Likewise, consistent with several studies that have shown that HA can improve the osteogenic potential of scaffolds (Frasca et al., 2017; Henriques Lourenço et al., 2017; Li et al., 2018; Wei et al., 2018), we found that the presence of HA further enhanced the regenerative capacity of DAT hydrogel, particularly when it was combined with OIASCs. HA is an inorganic component of bone, it has proven to be biocompatible and osteoinductive in nature, which makes it one of the most widely used materials in composite biomaterials for bone regeneration (Frasca et al., 2017).

Decellularized tissue-based scaffolds have also been tested previously for the treatment of critical-sized bone defects. Decellularized bone and periosteum in combination with BMSCs have displayed the ability to enhance regeneration of critical-sized long bone defects (Smith et al., 2014a; Kaempfen et al., 2015; Pennington et al., 2015; Ye et al., 2018). Although, the use of DAT for bone regeneration has not been reported before, Li et al. showed that intact adipose tissue in conjunction with OCT-4-Lv induced ASCs could promote regeneration of femoral defect (Li et al., 2013). Similarly, our results indicate that even after decellularization, adipose tissue in the form of DAT hydrogel maintained osteo-conductive capability. Although several previously reported studies based on critical-sized femoral defect model have used plates and external fixators to stabilize the defect (Alaee et al., 2014; Bougioukli et al., 2016), we chose to use intramedullary pin based stabilization method as described by Clough et al. (2015b), due to simplicity of the model and reproducibility of bone non-union in untreated control animals.

A limitation of the present study is that the biomechanics of regenerated bones were not analyzed. Biomechanical testing is a destructive procedure that would not have allowed other histological analyses, thus substantially increasing the number of animals required for the study. Additionally, a positive control group treated with autologous bone graft would have provided a comparison of the hydrogel vs. the current standard of care. However, the small size of mouse bones was a significant impediment in the formation of bone grafted positive control. BMSCs would also have provided an additional positive control based on their proven ability to enhance bone regeneration in earlier studies (Nau et al., 2018). However, to minimize the number of animals in the study we used ASCs only for the current experiments but will consider the use of BMSCs as positive control groups in future experiments. Furthermore, the surgical model employed, using a press fitted implant to stabilize the critical sized femoral defect, may be subject to some level of rotational instability upon ambulation. This could, in turn, lead to the localized formation of ectopic bone due to heterotopic ossification, a known complication in routine orthopedic surgeries (Hoyt et al., 2018). Similarly, rotational instability could also have resulted in pseudarthrosis which is visible in histological analysis (Figures 5, 6).

Finally, the current study was performed in immunocompetent C57BL/6 mice rather than immunodeficient nude or NOD/SCID/beige mice to allow for a more robust evaluation of the safety as well as the efficacy of human adipose-derived stromal cell transplants. While the current findings support both safety and efficacy, further analyses will be necessary to determine if the mice mounted a systemic immune response such as the generation of anti-human ASC antibodies. Future studies will be conducted to address immune responses mounted against individual or sequential xeno-transplantations of human ASC in immunocompetent murine models. This information will have potential relevance to the use of allogeneic human ASC transplantation in a clinical setting. In future ASC/DAT composite made from a larger donor pool must be tested to determine if donor-based variability or co-morbidities would affect the osteogenic potential of this scaffold. Furthermore, chemically modified forms of DAT [as previously described (Cheung et al., 2014; Brown et al., 2015; Pati et al., 2015)] could be tested to determine the optimum stiffness required for bone regeneration. Additionally, DAT hydrogel may be tested for the treatment of critical-sized defect in larger animals (e.g., pigs, dogs). The canine or porcine bones would allow for a more thorough investigation of bone biomechanics and would advance the clinical translation of DAT hydrogel-based therapy to human subjects.

## Conclusions

The present study presents DAT hydrogel as a potential alternative biomaterial for regeneration of critical-sized bone defects. DAT hydrogel is capable of regenerating critical-sized femoral defect in mice on its own, whereas the addition of OIASCs and HA augment its regenerative potential and result in the formation of a superior quality bone at a faster rate. In future, mechanical testing of regenerated bone could be performed to further strengthen these findings.

## Data Availability

The raw/processed data required for these findings can be made available by correspondence with Omair Mohiuddin (omohiudd@tulane.edu, omair.anwar@yahoo.com).

## Ethics Statement

The animal study was reviewed and approved by Tulane University Institutional animal care and use committee.

## Author Contributions

OM, JG, and BB conceived and designed the study. OM, BC, JP, MM, ER, DG, and MH collected and/or analyzed the data. OM wrote the manuscript. JG, BB, DH, and MH edited the manuscript.

### Conflict of Interest Statement

JG is affiliated with LaCell LLC, Obatala Sciences, and Talaria Antibodies. The remaining authors declare that the research was conducted in the absence of any commercial or financial relationships that could be construed as a potential conflict of interest.
