# Supplementary material for: Decellularized Adipose Tissue Hydrogel Promotes Bone Regeneration in Critical-Sized Mouse Femoral Defect Model
Source: Front Bioeng Biotechnol. 2019 Sep 6;7:211. doi: 10.3389/fbioe.2019.00211 (PMC6743019; doi:10.3389/fbioe.2019.00211)
Supplement: Supplementary file 1 [file Data_Sheet_1.PDF]

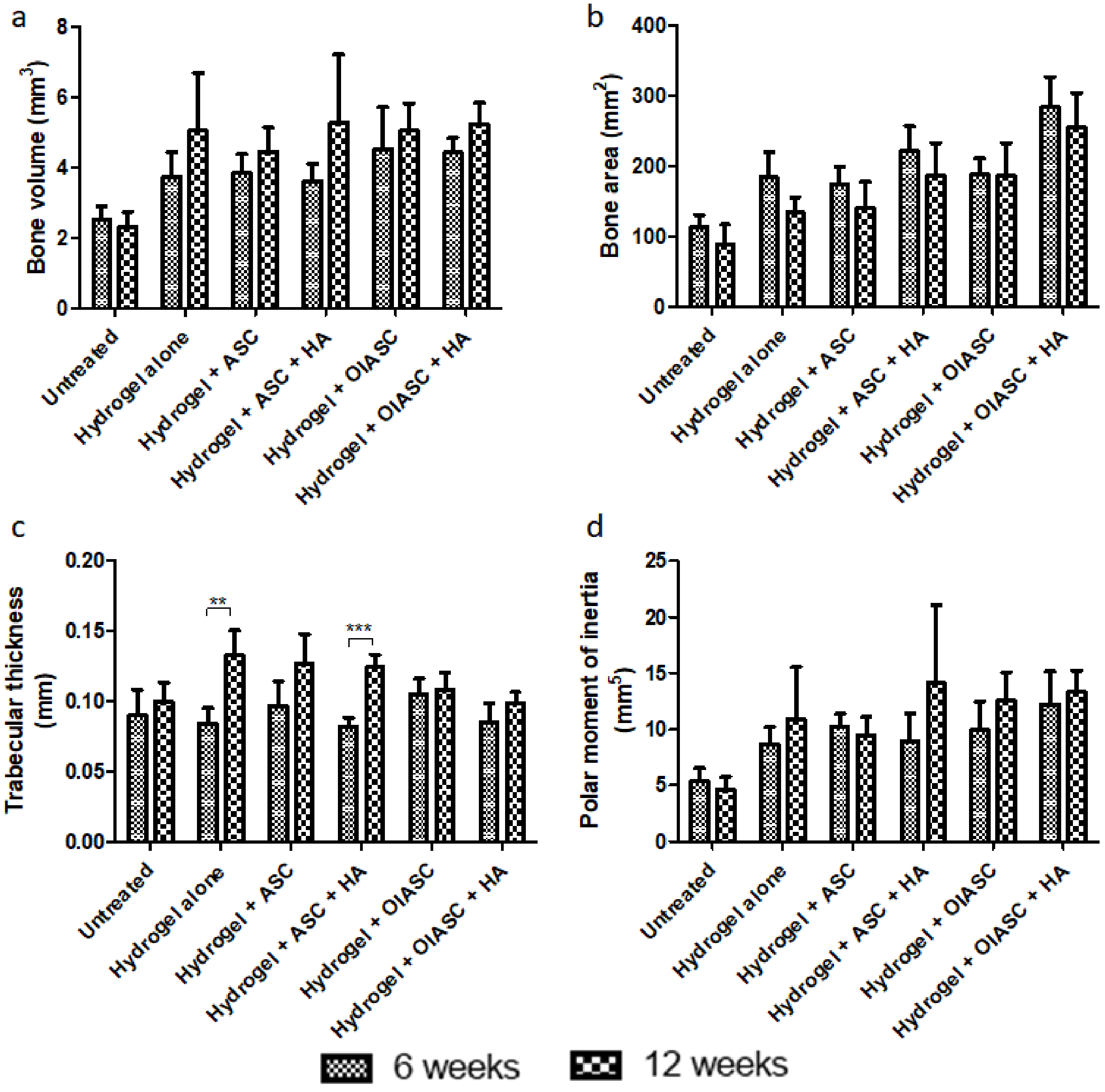

Figure S1: Comparative µCT analysis at 6 vs 12 week time point. Measurements were made of regenerated bone volume (a), bone area (b), trabecular thickness (c), and polar moment of inertia (d) using reconstructed axial µCT images Data are expressed as mean (n=4) ± SD; level of significance: (*) $p < 0.05$; (**) $p < 0.01$; (***) $p < 0.001$.

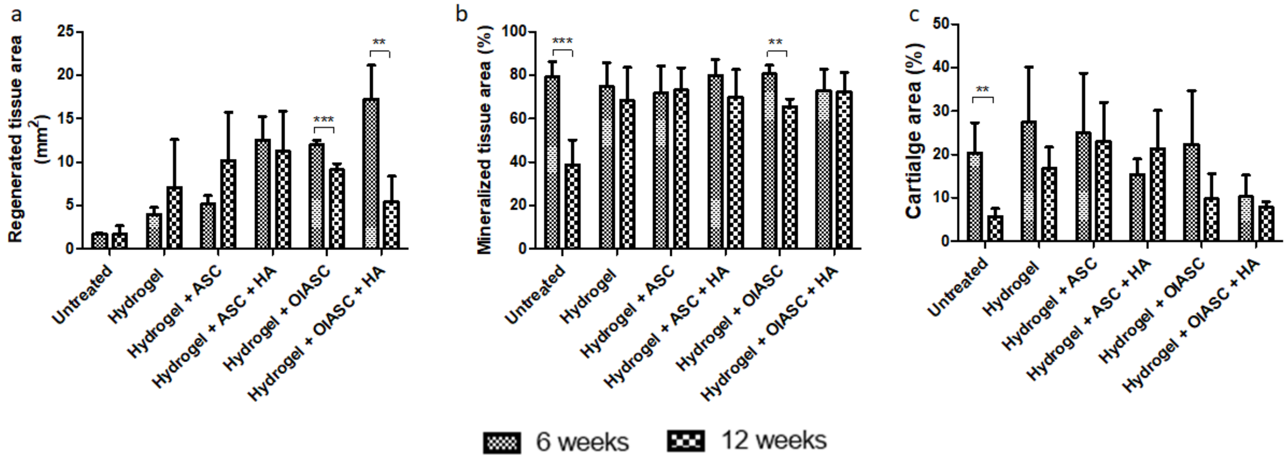

Figure S2: Comparative histological analysis at 6 vs 12 week time point. (a) Regenerated tissue area was determined by H&E stain, (b) percent mineralzied tissue area was determined by MT stain, and (c) percent cartilage area was determined by SO stain. Data are expressed as mean (n=4) ± SD; level of significance: (*) $p < 0.05$; (**) $p < 0.01$; (***) $p < 0.001$.

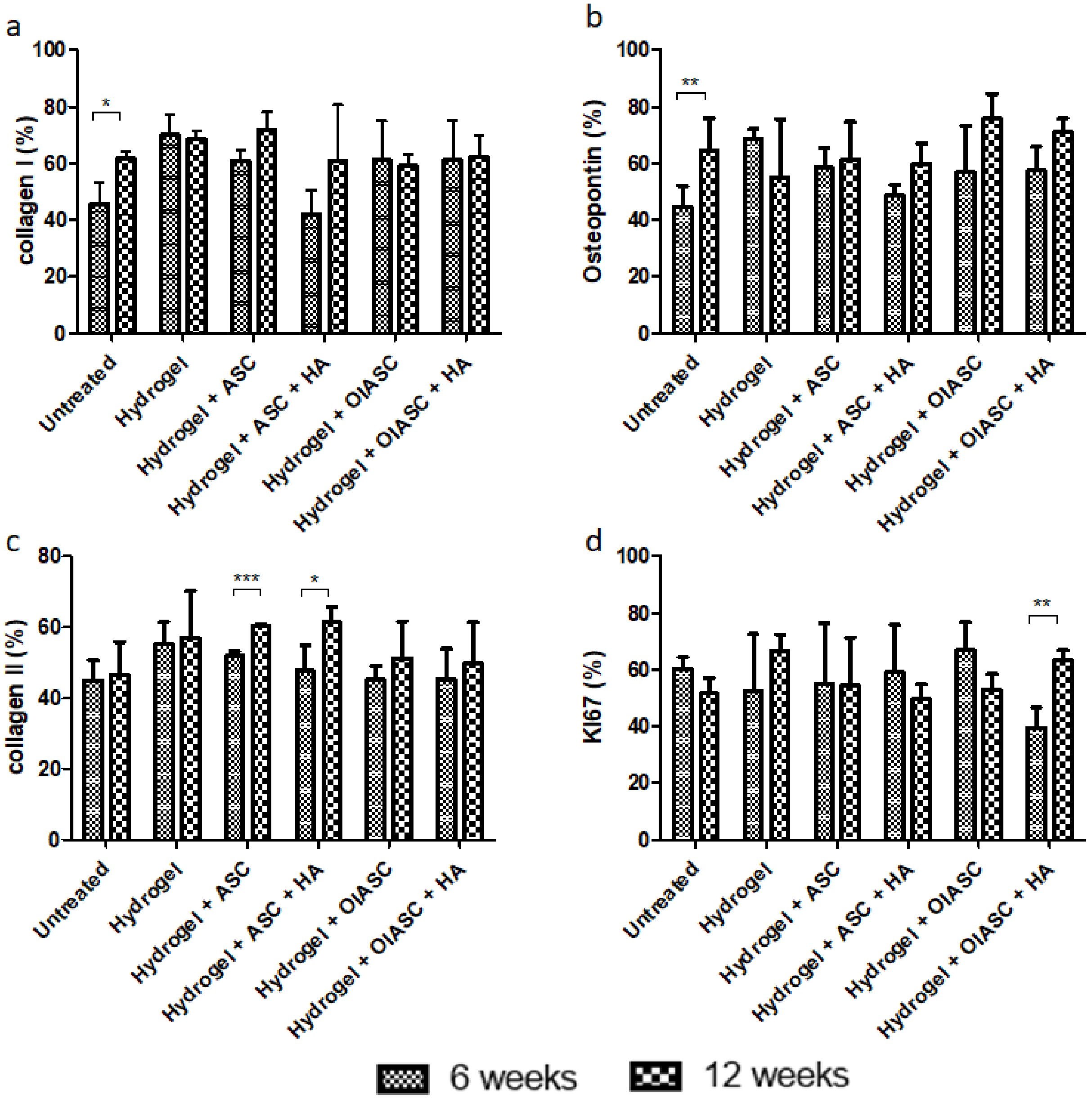

Figure S3: Comparative immunohistochemical analysis at 6 vs 12 week time point. Quantification of HRP/DAB signal was used to determine the area percentage of (a) collagen I, (b) osteopontin, (c) collagen II, and (d) osteopontin. Data are expressed as mean (n=4) ± SD; level of significance: (*) $p < 0.05$; (**) $p < 0.01$; (***) $p < 0.001$.
